# Identification of key Genes and Pathways Associated With Thermal Stress in Peripheral Blood Mononuclear Cells of Holstein Dairy Cattle

**DOI:** 10.3389/fgene.2021.662080

**Published:** 2021-06-10

**Authors:** Hao Fang, Ling Kang, Zaheer Abbas, Lirong Hu, Yumei Chen, Xiao Tan, Yachun Wang, Qing Xu

**Affiliations:** ^1^Institute of Life Sciences and Bio-Engineering, Beijing Jiaotong University, Beijing, China; ^2^Key Laboratory of Animal Genetics, Breeding and Reproduction, MARA, National Engineering Laboratory for Animal Breeding, Beijing Engineering Technology Research Center of Raw Milk Quality and Safety Control, College of Animal Science and Technology, China Agricultural University, Beijing, China

**Keywords:** thermal stress, PBMCs, differentially expressed genes, key genes, pathways, holstein dairy cattle

## Abstract

The objectives of the present study were to identify key genes and biological pathways associated with thermal stress in Chinese Holstein dairy cattle. Hence, we constructed a cell-model, applied various molecular biology experimental techniques and bioinformatics analysis. A total of 55 candidate genes were screened from published literature and the IPA database to examine its regulation under cold (25°C) or heat (42°C) stress in PBMCs. We identified 29 (3 up-regulated and 26 down-regulated) and 41 (15 up-regulated and 26 down-regulated) significantly differentially expressed genes (DEGs) (fold change ≥ 1.2-fold and *P* < 0.05) after cold and heat stress treatments, respectively. Furthermore, bioinformatics analyses confirmed that major biological processes and pathways associated with thermal stress include protein folding and refolding, protein phosphorylation, transcription factor binding, immune effector process, negative regulation of cell proliferation, autophagy, apoptosis, protein processing in endoplasmic reticulum, estrogen signaling pathway, pathways related to cancer, PI3K- Akt signaling pathway, and MAPK signaling pathway. Based on validation at the cellular and individual levels, the mRNA expression of the *HIF1A* gene showed upregulation during cold stress and the *EIF2A*, *HSPA1A*, *HSP90AA1*, and *HSF1* genes showed downregulation after heat exposure. The RT-qPCR and western blot results revealed that the *HIF1A* after cold stress and the *EIF2A*, *HSPA1A*, *HSP90AA1*, and *HSF1* after heat stress had consistent trend changes at the cellular transcription and translation levels, suggesting as key genes associated with thermal stress response in Holstein dairy cattle. The cellular model established in this study with PBMCs provides a suitable platform to improve our understanding of thermal stress in dairy cattle. Moreover, this study provides an opportunity to develop simultaneously both high-yielding and thermotolerant Chinese Holstein cattle through marker-assisted selection.

## Introduction

Extreme climatic events are the main threats to the survivability and maintenance of the dairy industry around the world (Das et al., 2016). The temperature at which cows show optimum production ranges from –5 to 25°C, once exceeds this range Holstein dairy cattle show signs of cold or heat stress (Berman et al., 1985; Bernabucci et al., 2010). Cold or heat stress can disrupt the physiological balance, and has negative effects on reproduction capabilities, milk quality, and yield, as well as the immune function of the dairy cows (Berman et al., 1985; West, 2003; Das et al., 2016; Abbas et al., 2020b). It has been reported that heat stress has caused an economic shortfall of $ 0.897 to $ 1.50 billion per year to the United States dairy industry (Stpierre et al., 2003; Baumgard and Rhoads, 2013). The severity of heat stress is expected to grow in the near future because of the progress in global warming, especially in subtropical, tropical, and arid parts of the world (Belhadj et al., 2016). It is also well established that ambient temperature lower than the normal shows detrimental effects on the production, health, and well-being of the dairy animals. In regions with limited agriculture such as North Asia, cold stress has emerged as a major impediment to the efficient growth of the livestock industry (Chebel et al., 2007; Angrecka and Herbut, 2016). Current mitigation strategies to cope with thermal stress in the dairy industry include nutritional management and physical adjustment of the external environment (Beede and Collier, 1986). However, the most economical and effective measure to deal with thermal stress is the selective breeding of both high-yielding and thermotolerant breeds (Renaudeau et al., 2012). This highlights the importance of elucidating the biological mechanism and the genetic factors of thermal stress response in dairy cattle.

Animals respond to thermal stress through evolutionarily conserved processes that regulate differential gene expression and related pathways (Feder and Hofmann, 1999; Kregel, 2002). Several studies have revealed numerous potential regulatory genes and pathways related to thermal stress in cattle using transcriptome analysis or microarray hybridization (Srikanth et al., 2017; Xu et al., 2017; Liu et al., 2020). In heat-stressed Holstein calves, 465 genes were significantly up-regulated and 49 genes were significantly down-regulated, and several biological processes and pathways were enriched including immune response, chaperones, apoptosis, protein processing in the endoplasmic reticulum, PI3K/AKT activation, estrogen signaling pathway, MAPK signaling pathway, among others (Srikanth et al., 2017). Another study reported 119 up-regulated and 81 down-regulated genes in Holstein dairy cows under heat stress (Liu et al., 2020), furthermore these genes were found to involve in several important pathways such as nucleotide excision repair, cAMP signaling pathway, antigen processing, and presentation. At present, there are few studies on cattle transcriptome related to cold stress. Previously we identified 193 differentially expressed genes (DEGs) in peripheral blood samples from Sanhe cattle after exposure to severe cold stress (–32°C for 3 h), which were involved in biological pathways, such as lipid metabolism and cell death and survival (Xu et al., 2017). There is growing evidence that thermal stress is associated with oxidative stress, endoplasmic reticulum stress, and neurochemical stress (Bernabucci et al., 2002; Lord-Fontaine and Averill-Bates, 2002; Mujahid et al., 2007; Srikanth et al., 2017; Alemu et al., 2018). Many genes of the heat-shock protein (HSP) families were involved in several regulatory pathways related to cellular thermal stress response and possess key cytoprotective effects (Feder and Hofmann, 1999). Although these findings contribute to a better understanding of biological mechanisms underlying the thermal stress response in cattle, changes in the expression of various genes that might be related to thermal stress have not yet been further validated at the cellular and individual levels. Additionally, the genes and pathways that play key roles in the acute thermal stress response and the interactions between them remain largely unknown which needs to be explored further.

The impact of thermal stress on cows is difficult to assess due to the complexity of animal metabolic and physiological systems. Peripheral blood mononuclear cells (PBMCs) are a common cell model that can be easily isolated and cultured to reflect the overall physiological condition of the cattle (Lacetera et al., 2006; Kishore et al., 2014; Kim et al., 2020). Therefore, the present study is designed to identify key genes and pathways that responded to acute thermal stress in Holstein dairy cattle at the PBMCs and individual levels. We screened candidate genes associated with thermal stress response in cattle by constructing an acute thermal stress cell model, applying various bioinformatics analysis and molecular biology experimental techniques. This work will further help improve our understanding of thermal stress response and may potentially be used as a reference for marker-assisted selection to develop both high-yielding and thermotolerant Holstein dairy cattle.

## Materials and Methods

### Screening Candidate Genes Associated With Thermal Stress in Cattle

**FIGURE 1:**
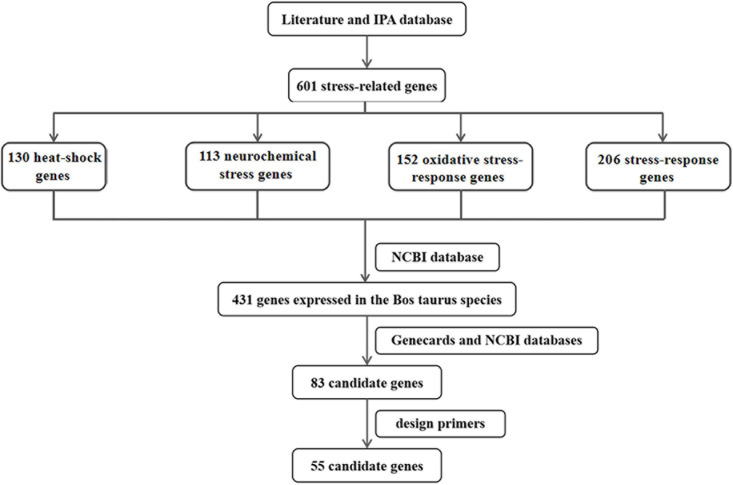
The flow chart of screening candidate genes related to thermal stress in cattle.

Thermal stress is associated with oxidative stress, endoplasmic reticulum stress, and neurochemical stress (Bernabucci et al., 2002; Lord-Fontaine and Averill-Bates, 2002; Mujahid et al., 2007; Srikanth et al., 2017; Alemu et al., 2018). In this study, 601 stress-related genes were searched by keywords from the published literature and the IPA database, including 130 heat-shock genes, 113 neurochemical stress genes, 152 oxidative stress-response genes, and 206 stress-response genes (Figure 1). After removing duplicate genes between different categories, a sum of 431 genes expressed in Bos taurus species were identified from the NCBI database^1^ and the function of the 431 genes was searched from the Genecards database^2^. Referring to Genecards and NCBI databases, we screened genes that were highly correlated with thermal stress-response and highly expressed in the blood. Finally, a total of 83 candidate genes associated with thermal stress response in the Bos taurus species were screened. However, primers of 55 candidate genes (34 heat-shock genes, 19 neurochemical stress genes, 11 oxidative stress-response genes, 19 stress-response genes, and 12 affecting stress-response genes) and 1 internal reference gene (*GAPDH*) were successfully designed for RT-qPCR using Primer3 web version 4.0.0^3^ and Primer blast^4^ The sequences of the primers were shown in Supplementary Table 1. Therefore, these 55 candidate genes were used for subsequent research.

### Peripheral Blood Mononuclear Cells (PBMCs) Isolation by Using Ficoll-Paque Density Gradient Centrifugation

Holstein dairy cattle peripheral venous blood was collected from Beijing Sanyuan dairy farm, and the PBMCs were isolated from these blood samples at room temperature within 4 h by using the Ficoll-Paque density gradient centrifugation. Briefly, ACD-A-anticoagulated blood samples were centrifuged at 400 × *g* for 10 min to remove the upper layer of plasma. The remaining blood cells were diluted with an equal volume of 1 × phosphate-buffered saline (PBS, pH 7.4), which contains 0.05 M ethylenediaminetetraacetic acid (EDTA, Invitrogen, Carlsbad, CA); and then 12.5 mL of diluted blood was carefully coated over 25 mL of the Ficoll-Paque PLUS (GE Healthcare, Piscataway, NJ) to produce a clear boundary. Then, gradients were centrifuged at room temperature at 400 × *g* for 40 min in a swinging bucket rotor with no brake application. The white opaque mononuclear fraction was carefully removed to a new centrifuge tube by pipetting and washed with 5 times volume of PBS-EDTA by centrifugation at room temperature at 200 × *g* for 20 min. Lastly, the cells were washed three times in 1 × PBS (pH 7.4) to obtain PBMCs.

### Cell Culture and Acute Thermal Stress Treatment

The PBMCs culture was prepared in RPMI 1640 medium (Gibco, Los Angeles, CA) comprising 1% penicillin-streptomycin and 10% fetal bovine serum (FBS, Gibco, Los Angeles, CA), and were cultured in a humidified atmosphere of 95% air and 5% CO_2_ at 37°C.

Since Holstein cattle are cold but not heat tolerant (Thornton et al., 2009), based on relevant references (Underhill and Smales, 2007; Neutelings et al., 2013; Kishore et al., 2014; Li et al., 2015; Khan et al., 2020), 10, 25, and 32°C were chosen for cold stress treatment, while 39 and 42°C were chosen for heat stress treatment, and 37°C treatment group was the control group. After incubation in 12 or 96-well plates at 37°C for 24 h, PBMCs were then exposed to different temperatures for 1 h in an isothermic water bath incubator (Jing Hong Laboratory Instrument Co., Ltd., Shanghai, China). Finally, PBMCs in 12-well plates were then harvested to extract total RNA or protein for RT-qPCR or western blot to detect the expression of inducible *HSPA1A*, and PBMCs in 96-well plates were harvested to assay cell viability.

### Cell Viability Assay After Acute Thermal Stress

After treatment with different temperatures for 1 h, according to the manufacturer’s protocols, the cell viability of PBMCs was assayed by the MTS test (Cell Titer96; PROMEGA. Madison, WI, United States). A 20 μL working solution was added to each well of the 96-well plates containing cell culture medium. These 96-well plates were then shielded with foil to protect from light-rays and incubated in 5% CO_2_ at 37°C for 4 h. When the incubation was finished, the optical density was measured at 490 nm in a microplate reader (Multiskan FC; type 357, Thermo Scientific, Waltham, MA, United States). The test was assayed five times under the same conditions.

### Animals Selection and Peripheral Blood Leukocytes Sampling

The animal study was reviewed and approved by the Committee on Ethics of Animal Experimentation from the Beijing Jiaotong University, Beijing, China (Code ID: SS-QX-2014-06). In summer (August 4th, 2015), autumn (October 31th, 2015), and winter (January 10th, 2016), 20 Holstein dairy cattle with consistent feeding and management, same parity, and similar body condition were selected from Beijing Sanyuan dairy farm. These 20 individuals were born from 2 sires, and there is no selection against thermal stress in the sire of these cows. The average number of days in milk (DIM) on the sampling day for these 20 Holstein dairy cattle was 62 (in summer), 151 (in autumn), and 221 (in winter) days, respectively. Their average 7-day milk yield before the sampling day was 37 kg/d (in summer), 36 kg/d (in autumn), and 31 kg/d (in winter), respectively. The average ambient temperature and THI calculated 1 week before sampling were summer (30.52°C, THI = 80.04), autumn (16.01°C, THI = 59.53), winter (7.13°C, THI = 49.33); while the ambient temperature and THI during sampling were summer (32.51°C, THI = 81.8), autumn (16.24°C, THI = 60), winter (2.58°C, THI = 43.5). Peripheral venous blood (10 mL) from each cow was collected through the coccygeal vein into the EDTA tube. These blood samples were stored at room temperature for 1 h and then centrifuged at 3,000 × rpm for 15 min. The white blood cells were immediately separated into new tubes, after adding 1 mL Trizol (Invitrogen, Carlsbad, CA), samples were frozen at –20°C and then stored at –80°C before RNA isolation.

### RNA Isolation of PBMCs and Peripheral Blood Leukocytes

Trizol reagent (Invitrogen, Carlsbad, CA) was used according to the manufacturer’s protocols to separate total RNA from PBMCs of different temperature treatment groups and white blood cell samples of different seasons, respectively. Subsequently, the RNeasy kit (Qiagen, Valencia, CA) was used to eliminate the remaining genomic DNA. Finally, the total RNA was eluted in RNase-free water, Nanodrop 2000 spectrophotometer (Thermo Scientific, Wilmington, DE) was used to quantify, and Agilent 2100 Bioanalyzer (Agilent Technologies Inc., Santa Clara, CA) was used to assess the quality. The RNA samples with the 260/280 ratio greater than 1.8 and the RNA integrity numbers greater than 8.0 were considered to meet the purity criteria.

### Quantitative Reverse Transcription PCR (RT-qPCR)

The RT-qPCR analysis was used to detect the mRNA expression changes of the *HSPA1A* gene, HSP family genes, and other candidate genes in cellular and individual levels after thermal stress. Total RNA was reverse transcribed using a first-strand cDNA synthesis Kit (Thermo Fisher Scientific, Germany) with oligo (dT) 18 primers according to the manufacturer’s protocols. The RT-qPCR was conducted using iTaq^TM^ Universal SYBR^®^ Green Supermix (Bio-Rad Laboratories GmbH, Germany) in Applied Biosystem^®^ StepOnePlus^TM^ (Applied biosystems, CA, United States). Amplification was conducted in a 20 μL reaction volume with 2 μL of cDNA (10 ng/μL), 10 μL of 1 × SYBR Green Master Mix (Bio-Rad Laboratories GmbH, Germany), 0.6 μL of forwarding primers, 0.6 μL of reverse primers (Supplementary Table 1), and 6.8 μL of RNase-Free H_2_O. The *GAPDH* gene was used to standardize the relative abundance of genes. We analyzed all data from three technical replicates for each sample by using the 2^–ΔΔCt^ method (Livak and Schmittgen, 2001).

### Bioinformatics Analysis for Differentially Expressed Genes (DEGs)

Based on the database for Annotation, Visualization and Integrated Discovery (DAVID) version 6.8^5^ (Huang et al., 2009), the gene ontology (GO) functional annotation analyses were performed to categorize DEGs, including biological processes (BP), cellular components (CC), molecular function (MF) terms and Kyoto Encyclopedia for Genes and Genomes (KEGG) pathway analysis. The cut-off point was delimited as *P* < 0.05 and Benjamini-Hochberg false discovery rate (FDR) < 0.05. We used the Search Tool for the Retrieval of Interacting Genes (STRING) version 10.5^6^ to construct the protein-protein interaction (PPI) networks. The PPI network was visualized using Cytoscape (version 3.8.1)^7^.

### Screening Important Genes Associated With Thermal Stress

We defined genes that were significantly associated with thermal stress based on the following criteria: (1) mRNA expression changes of genes were large (fold change ≥ 1.2-fold) and significant (*P* < 0.05) after thermal stress, the mRNA expression of HSP families genes should be changed significantly and largest in their families; (2) genes involved in important functions and pathways in response to thermal stress in GO functional annotation and KEGG pathway enrichment analysis; (3) genes corresponding proteins act as hub proteins that could interact with different kinds of proteins in the PPI network.

### Western Blot Analysis

The western blot was performed to detect changes in the protein expression of the *HSPA1A* gene and other genes at the cellular level after exposure to thermal stress. After harvested, the PBMCs were lysed immediately in radioimmune precipitation assay (RIPA) buffer (Beyotime, Shanghai, China), the bicinchoninic acid (BCA) assay (Thermo Fisher Scientific, Germany) were used to measure the protein concentrations. After denatured at 100°C for 10 min, equal amounts of total protein were separated by SDS-PAGE (12% acrylamide gel containing 0.1% SDS), and then the protein bands were transferred onto PVDF membranes (BioTraceNT, Pall Corp., Port Washington, NY, United States). After blocked with 5% skimmed milk in Tris-buffered saline (TBS) containing 0.1% Tween 20 (TBST) for 1 h at 37°C, the membranes were then incubated with primary antibodies against DNAJB1 (1:1,000, Abcam, Cambridge, MA, United States), EIF2A (1:1,000, Abcam, Cambridge, MA, United States), HIF1A (1:1,000, Abcam, Cambridge, MA, United States), HSPA1A (1:1,000, Santa Clara, CA), HSP90AA1 (1:1,000, Abcam, Cambridge, MA, United States), HSPD1 (1:1,000, Abcam, Cambridge, MA, United States), HSF1 (1:1,000, Santa Clara, CA), and β-actin (1:5,000, Sigma, MO, United States) at 4°C overnight after washing three times in TBST, followed by incubation with polyclonal HRP-conjugated secondary antibodies (1:3,000, GBI, WA, United States) at room temperature for 1 h. The protein bands were then visualized by enhanced chemiluminescence (ECL) and analyzed by densitometry using the NIH Image J software (version 1.46), using the β-actin protein as the loading control.

### Statistical Analysis

The statistical analyses were performed using SAS 9.2 software. Statistical differences between two groups were tested by two-sided student’s *t*-test, and statistical differences between three or more groups were analyzed by one-way analysis of variance (ANOVA). The ^∗^*P* < 0.05 or the ^∗∗^*P* < 0.01 were considered to indicate significant differences. Results were presented as the mean ± standard deviation from at least three independent experiments.

## Results and Discussion

### Construction of the Acute Thermal Stress Cell Model

**FIGURE 2:**
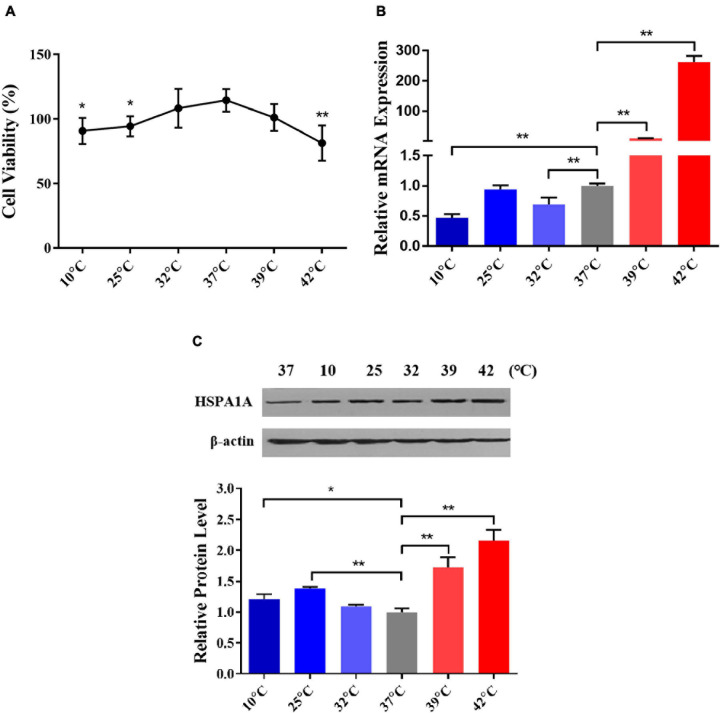
25 and 42°C were selected as the treated temperatures for acute cold and heat stress in PBMCs, respectively. **(A)** The changes in PBMCs cell viability after different temperatures treated for 1 h were assayed by the MTS test. **(B)** The relative mRNA expression changes of the *HSPA1A* gene in PBMCs after diverse temperatures treated for 1 h were analyzed by RT-qPCR. **(C)** The relative protein expression changes of the *HSPA1A* gene in PBMCs after different temperatures treated for 1 h were detected by western blot. The error bar denotes the standard deviation of the mean (*n* = 3). Statistical analyses were performed by one-way ANOVA, the results only showed the comparison between each treatment group and control group (37°C). *p < 0.05 and **p < 0.01.

To select the optimum cold and heat stress treatment temperatures, we comprehensively considered the cell viability and the expression of inducible *HSPA1A* gene (a member of the HSP70 family) in PBMCs after treated at different temperatures viz. 10, 25, 32, 37, 39, and 42°C for 1 h. In comparison to the control (37°C), the cell viability of PBMCs declined in temperature-dependent fashion, and declined significantly (*P* < 0.05) after treatment with 10, 25, and 42°C for 1 h (Figure 2A). This indicates that the damage to the cells was related to the degree of thermal stress. Similar results were also found in HeLa cells and riverine buffaloes PBMCs (Huang et al., 2009). By using RT-qPCR and western blot, the relative abundance of *HSPA1A* at mRNA and protein levels was detected, respectively. Our results indicated that in comparison to the control group (37°C), the mRNA expression of the *HSPA1A* gene, although non-significant, increases after treatment with 25°C for 1 h (Figure 2B); the protein expression of HSPA1A was significantly up-regulated (*P* < 0.01) and reached the highest levels after treatment with 25 and 42°C for 1 h, respectively (Figure 2C), which was consistent with the results of cell viability. Therefore, according to the above results, 25 and 42°C were selected as the standard temperatures for acute cold and heat stress in Holstein dairy cattle PBMCs for subsequent experiments.

Hsp70 is one of the most conserved, sensitive, and abundant gene related to stress response among the HSP family genes (Kiang and Tsokos, 1998; Manjari et al., 2015), increased expression of HSP70 family genes indicate that cells initiate self-protection mechanisms in response to environmental stress, and could be used as biomarkers for the evaluation of bovine physiology and cellular thermal stress response (Sonna et al., 2002). As an essential member of the HSP70 family, the important roles of the *HSPA1A* gene in regulating thermal stress response have been identified (Deane and Brown, 2018; Bilog et al., 2019). Besides, our previous studies have found that Sanhe cattle after exposure to extreme cold stress, only *HSPA1A* expression was upregulated among 27 genes encoding HSP-binding proteins, heat shock transcription factors, and heat shock proteins (Xu et al., 2017). The expression of *HSPA1A* was related to the levels of blood hormones (Hu et al., 2019; Abbas et al., 2020a). Consistent with our present study, 25°C was already adopted as the mild cold shock treatment temperature to study cold stress response in WI26 cells (Neutelings et al., 2013), whereas 42°C was adopted to simulate heat stress conditions for Korean native male beef calves (Kim et al., 2020), riverine buffaloes (Bubalus bubalis), exotic cattle (Bos taurus), and native cattle (Bos indicus) of India (Kishore et al., 2014). Hence, we selected 25 and 42°C to construct a feasible acute thermal stress cell model.

### Identification of Differentially Expressed Genes (DEGs)

**FIGURE 3:**
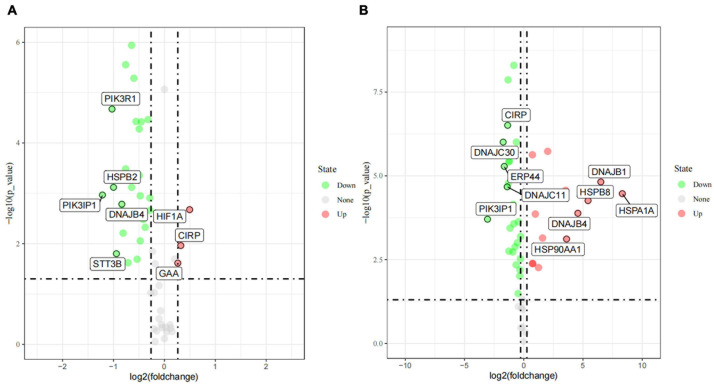
The differently expressed genes (DEGs) after acute cold **(A)** and heat stress **(B)** in PBMCs. Statistical analyses were performed by the student’s *t*-test, the 37°C treatment group was the control group; DEGs were defined with *FC* ≥ 1.2-fold and *p* < 0.05. If the number of DEGs is less than 5, all the gene names are displayed, and if it is greater than 5, only the names of the top five genes with the largest fold change are displayed.

**FIGURE 4:**
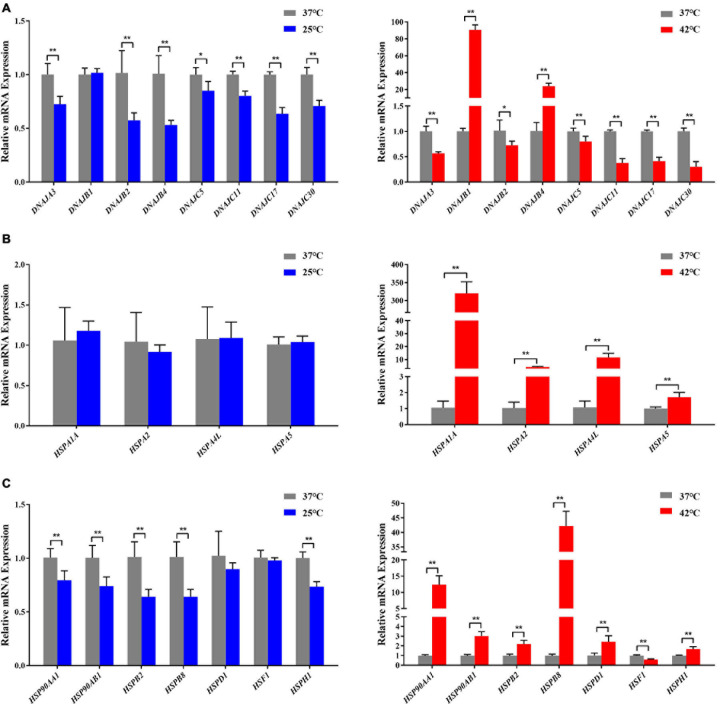
Acute thermal stress caused changes in the mRNA levels of candidate genes from HSP 40 family **(A)**, HSP 70 family **(B)**, other HSP families (HSP60, HSP90, HSP110, and sHSP) **(C)** in Holstein dairy cattle PBMCs. The error bar denotes the standard deviation of the mean (*n* = 3). Statistical analyses were performed by the Student’s *t*-test *p < 0.05 and **p < 0.01, in comparison to the control group (37°C).

As described in details above, 55 candidate genes related to thermal stress (34 heat-shock genes, 19 neurochemical stress genes, 11 oxidative stress-response genes, and 31 stress-response genes) were screened from relevant published literature and databases, and primers were successfully designed. According to the determined treatment temperatures for cold and heat stress, PBMCs were treated at 25, 37, and 42°C for 1 h, respectively. The RT-qPCR was used to analyze 55 candidate genes’ transcriptional expression differences among the three groups to identify DEGs. Under the assumptions of FC ≥ 1.2-fold and *P* < 0.05, a sum of 29 DEGs (Figure 3), comprising 3 (10.3%) up-regulated genes and 26 (89.7%) down-regulated genes were identified by comparing control vs. 25°C group. Among them, the 3 up-regulated DEGs were *HIF1A*, *CIRP*, *GAA*, and the top 5 down-regulated DEGs were *PIK3IP1*, *PIK3R1*, *HSPB2*, *STT3B*, *DNAJB4*. Similarly, compared with the control group, a sum of 41 DEGs (Figure 3) with 15 (36.6%) up and 26 (63.4%) down-regulated genes were identified in the 42°C group. Among them, the top 5 up-regulated DEGs were *HSPA1A*, *DNAJB1*, *HSPB8*, *DNAJB4*, *HSP90AA1*, while *PIK3IP1*, *DNAJC30*, *ERP44*, *DNAJC11*, and *CIRP* were the top 5 down-regulated DEGs. Cold stress leads to alter the cellular membranes’ lipid composition, decrease protein synthesis and metabolic rate, as well as suppress cell proliferation (Fujita, 1999), hence our results revealed that most candidate genes were down-regulated after acute cold stress (Figures 3, 4). Consistent with previous studies (Fujita, 1999), CIRP (cold-inducible RNA-binding protein), the first-ever identified cold shock protein in mammal cells, was induced after acute cold stress and down-regulated after acute heat stress.

In our present study, the RT-qPCR results confirmed that most HSP genes respond to acute thermal stress, especially when exposed to acute heat stress (Figures 3, 4). Among which *DNAJB1* of HSP40 family (Figure 4A), *HSPA1A* of HSP70 family (Figure 4B), *HSP90AA1* of HSP90 family, and *HSPB2*, *HSPB8*, *HSPD1*, *HSF1*, *HSPH1* of other HSP families (Figure 4C) had the largest (*P* < 0.05) expression changes at the cellular transcription level, respectively. The HSP families play important roles in response to environmental stress, most genes of the heat-shock protein (HSP) families have been investigated to participate in numerous regulatory pathways related to cellular thermal stress response and possess key cytoprotective effects (Feder and Hofmann, 1999). Our results are supported by some of the findings of previous studies in which heat stress stimulated the expression of HSP genes in many cell/tissue types including lymphocytes in buffalo (Mishra et al., 2011), bovine granulosa cells (Mishra et al., 2011), bovine endometrial tissue (Malayer et al., 1988), and bovine conceptuses (Putney et al., 1988).

### GO Functional Annotation and KEGG Pathway Enrichment Analyses of DEGs

**FIGURE 5:**
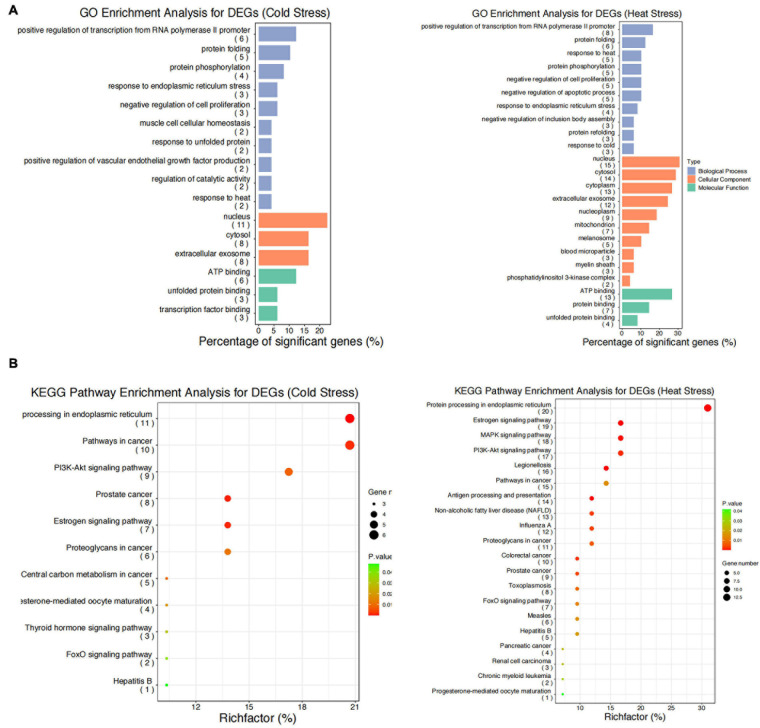
GO functional annotation and KEGG pathway enrichment analysis for the DEGs. **(A)** GO functional annotation for DEGs after acute cold and heat stress. **(B)** KEGG pathway enrichment analysis for DEGs after acute cold and heat stress. Summaries of Gene Ontology (GO) analysis were present in three categories: biological process, cellular component, and molecular function. Gene number: number of genes in each pathway.

We performed GO functional annotation and KEGG pathway enrichment analyses to identify the biological functions of the DEGs. The GO analysis exhibited that the DEGs were significantly (*P* < 0.05) enriched in biological processes including positive regulation of transcription from RNA polymerase II promoter, response to cold and heat stress, protein folding/re-folding, protein phosphorylation, negative regulation of cell proliferation and apoptotic process, and response to endoplasmic reticulum stress (Figure 5A). Meanwhile, for cellular components, the DEGs were primarily located in the nucleus, cytosol, extracellular exosome, mitochondrion, and melanosome (Figure 5A). Furthermore, it was found that molecular functions were enriched in ATP binding, protein and unfolded protein binding, transcription factor binding (Figure 5A). The KEGG pathway enrichment analyses showed that after acute thermal stress, the DEGs were markedly (*P* < 0.05) enriched in several processes including protein processing in endoplasmic reticulum, estrogen signaling pathway, antigen processing and presentation, PI3K- Akt signaling pathway, MAPK signaling pathway, pathways in cancer (Figure 5B and Supplementary Table 3).

**FIGURE 6:**
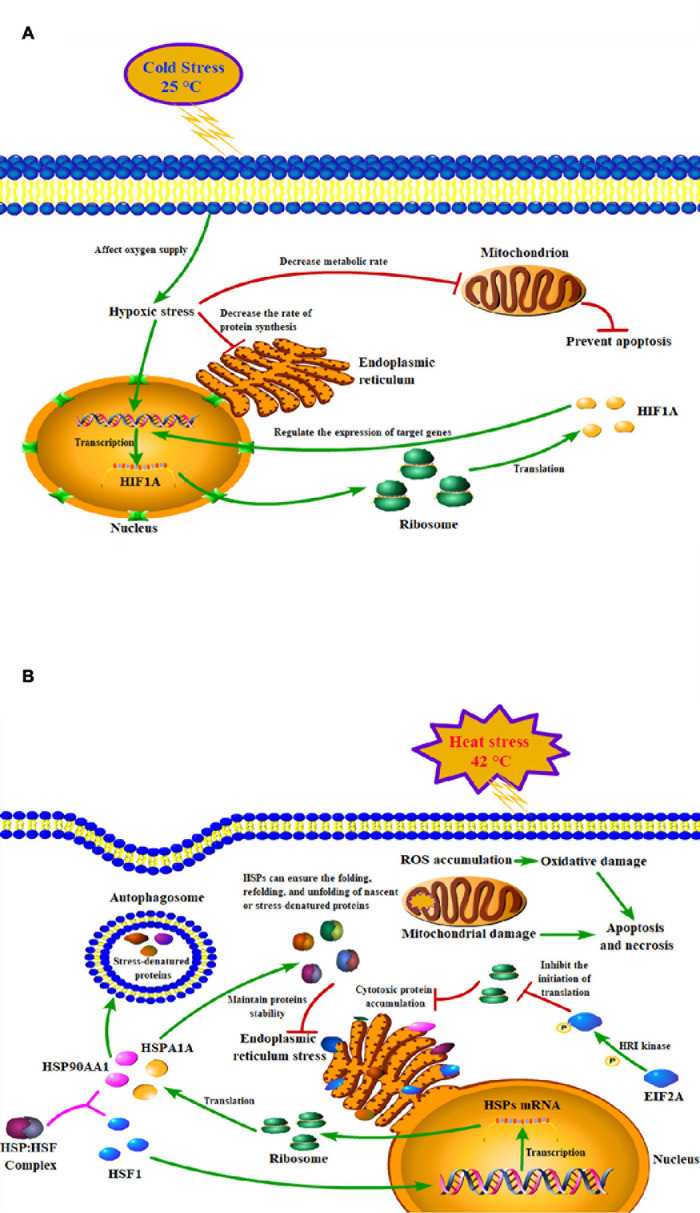
Models of the mechanisms of key genes in cold **(A)** and heat **(B)** stress responses of peripheral blood mononuclear cell.

The findings of our study suggested that acute heat stress lead to the activation of heat shock factors (HSF) and factors involved in protein folding/re-folding (Figure 3 and Supplementary Table 3). Heat stress causes a large amount of cytotoxic protein to accumulate in the endoplasmic reticulum, thereby triggering endoplasmic reticulum stress (Belhadj et al., 2016). Increased HSP family genes expression can ensure the folding, refolding, and unfolding of nascent or stress-denatured proteins and maintain proteins stability in response to thermal stress. They can also help protein processing in the endoplasmic reticulum, thus relieving endoplasmic reticulum stress-induced by heat stress (Figure 6B; Neuer et al., 1999; Lamoureux et al., 2014; Alemu et al., 2018).

Since acute cold stress decreases the rate of protein synthesis and metabolic rate, and suppresses cell activity (Fujita, 1999), most of the DEGs enriched in the pathways were down-regulated except *HIF1A* which was significantly upregulated (*P* < 0.05). Some studies have shown that after exposure to low temperatures, it altered microcirculation and affects oxygen supply to tissues thus leading to hypoxic stress and increased expression of hypoxia-inducible factor-1A (*HIF1A*), which acts as a transcription factor, and that hypoxia increases the stability of the HIF1A protein, which can enter to the nucleus and regulate the expression of target genes (Figure 6A; Gordon, 2001; Jain et al., 2013). A study has reported that the hypoxia/HIF1a signaling pathway can promote the progression of prostate cancer and it could be a new therapeutic target (Tran et al., 2020).

Thermal stress can also affect autophagy and apoptosis (Yang et al., 2016; Hale et al., 2017). Cells protect themselves during cold stress by preventing apoptosis via inhibiting DNA damage and mitochondrial dysfunction (Diestel et al., 2011). While heat stress can induce mitochondrial damage and oxidative stress to disturb the steady-state concentration of reactive oxygen species (ROS), which causes oxidative damage to the cells and induces apoptosis and necrosis pathways (Figure 6B; Jahan et al., 2019). PI3K/Akt signaling pathway is important autophagy- and apoptosis-related pathway that mediates physiological functions like cell proliferation, migration, cell differentiation, autophagy, and apoptosis (Levine and Kroemer, 2008), and it has been reported that this pathway played an important role in preventing apoptosis induced by heat stress (Gao et al., 2013). Our results found that genes of the HSP90 family may play key roles through the PI3K/Akt signaling pathway (Supplementary Table 3). Studies have shown that HSP90AA1 increased chemoresistance in osteosarcoma cells by inhibiting apoptosis and inducing autophagy through the PI3K/Akt/mTOR pathway (Xiao et al., 2018). Similarly, through the HSP90AA1-AKT-MTOR pathway, HSP90AA1 could also induce autophagy in early avibirnavirus infection (Hu et al., 2015).

Our results also indicated that genes of HSP70 and HSP90 families may play key roles in some pathways including immune-related response, MAPK signaling pathway, and estrogen signaling pathway (Supplementary Table 3). Thermal stress has serious effects on animal health and damages the immune function, which makes animals susceptible to pathogens (Strong et al., 2015). HSP70 can bind to TLR2 and TLR4 and induce the release of cytokines and chemokines and other immunoregulatory effects through a process called chaperone activity (Ju et al., 2014). The MAPK signaling pathway is implicated in inhibiting apoptosis and promoting cell growth (Wada and Penninger, 2004) and is involved in sperm damage induced by heat (Rahman et al., 2014), and is considered to be the main cause of infertility in livestock due to heat stress (Hansen, 2009).

Our results provide evidences that the above biological processes and pathways play key roles in thermal stress response in Holstein dairy cattle.

### Construction of Protein-Protein Interaction (PPI) Networks for DEGs

**FIGURE 7:**
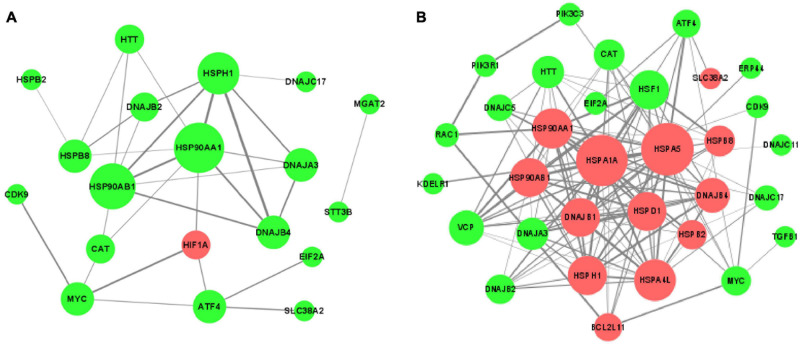
The protein-protein interaction (PPI) networks of DEGs corresponding proteins **(A)** after acute cold stress and **(B)** after acute heat stress. The nodes are proteins, and the edges are the interaction between the nodes. The larger of the nodes means the more proteins interact with them. The darker color of the edges means the stronger interaction between the two proteins. The red nodes show up-regulated DEGs, while the green nodes symbolize down-regulated DEGs.

In order to understand thoroughly the interconnection between the DEGs, the PPI networks among the corresponding proteins of DEGs were constructed by using the STRING online database (Figure 7). It was apparent from the figure that some proteins were within the PPI networks and could be considered as hub proteins: in the acute cold stress group, the corresponding proteins with the number of interactions greater than 3 were HSPAA1, HSP90AB1, HSPH1, DNAJB4, HSPB8, HIF1A, and DNAJB2; while for the acute heat stress group, the corresponding proteins with the number of interactions greater than 10 were HSPA5, HSPA1A, HSP90AA1, HSF1, DNAJB1, HSPD1, HSP90AB1, and DNAJB4. Our results also indicated that when exposed to acute thermal stress, the HSPs and chaperone molecules were closely related and mostly co-expressed to perform functions, like the ATPase activity of HSP70 was regulated by the interaction of HSP40 with its J domain (Wong, 1999); HSP70 and HSP90 proteins can be reversibly linked together by chaperones to form HSP70-HSP90 organizing protein (HOP) (Odunuga et al., 2004).

### Identification of Key Genes That Respond to Thermal Stress of Holstein Dairy Cattle

Based on the results and criteria that we described in details above, a total of 7 genes were recognized as important genes that respond to thermal stress, viz. *DNAJB1*, *EIF2A*, *HIF1A*, *HSPA1A, HSP90AA1*, *HSPD1*, and *HSF1.* Among them, *EIF2A*, *HIF1A*, *HSP90AA1* may play important roles that respond to cold stress, and all of the seven genes may play key roles in heat stress response.

**FIGURE 8:**
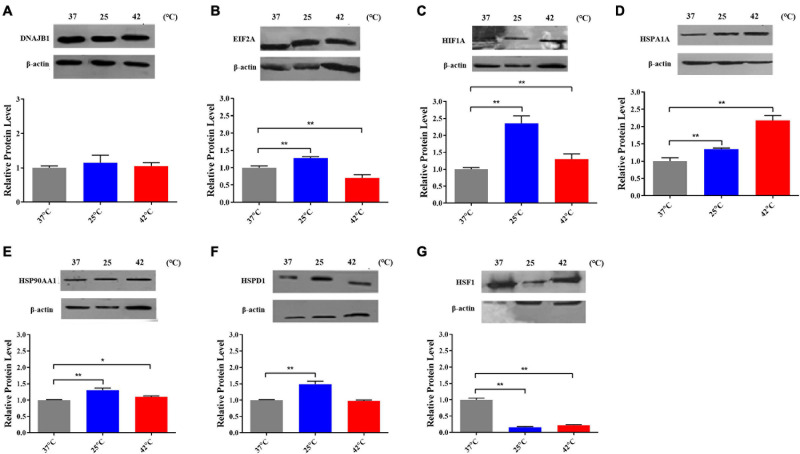
The proteins expression of **(A)** DNAJB1, **(B)** EIF2A, **(C)** HIF1A, **(D)** HSPA1A, **(E)** HSPAA1, **(F)** HSPD1, and **(G)** HSF1 after acute thermal stress in Holstein dairy cattle PBMCs was detected by western blot. The error bar denotes the standard deviation of the mean (*n* = 3). Statistical analyses were performed by one-way ANOVA, the results only showed the comparison between each treatment group and control group (37°C). *p < 0.05 and **p < 0.01.

**FIGURE 9:**
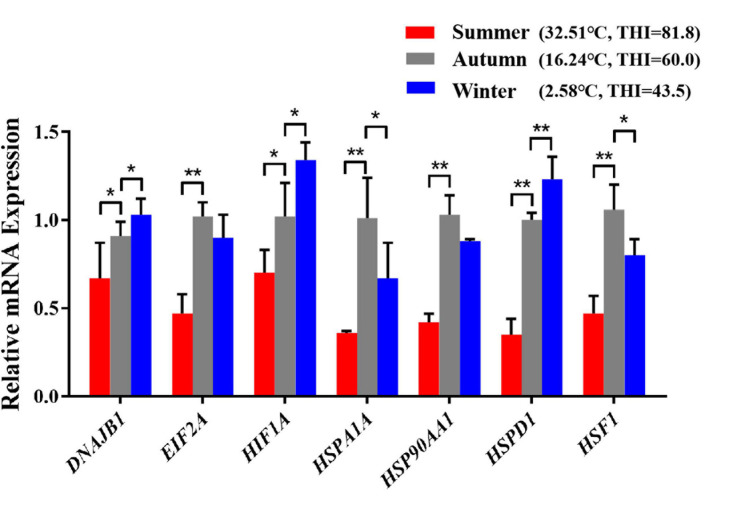
The differences in mRNA expression levels of important genes that respond to thermal stress in Holstein dairy cattle peripheral venous blood samples from different seasons. The average maximum ambient temperature and THI for the week before sampling were autumn (16.01°C, THI = 59.53), summer (30.52°C, THI = 80.04), winter (7.13°C, THI = 49.33); the maximum ambient temperature and THI for the sampling day were autumn (16.24°C, THI = 60), summer (32.51°C, THI = 81.8), winter (2.58°C, THI = 43.5). The error bar denotes the standard deviation of the mean (*n* = 3). Statistical analyses were performed by one-way ANOVA, the results only showed the comparison between each treatment group and control group (37°C). *p < 0.05 and **p < 0.01.

After exposed to acute cold stress and heat stress, western blot analysis was performed to detect the expression changes of the seven important genes at the cellular translation level (Figure 8). Combined with the previous RT-qPCR results (Figure 3), the *HIF1A* gene after cold stress and the *EIF2A, HSPA1A, HSP90AA1, HSF1* genes after heat stress had consistent trend changes at the cellular transcription and translation levels of Holstein dairy cattle PBMCs. The relative mRNA expression changes of the 7 important genes in Chinese Holstein dairy cattle peripheral venous blood samples from summer, autumn, and winter were detected by RT-qPCR (Figure 9), compared with autumn (non-thermal stress), the expression of the *HIF1A* gene in winter and the *EIF2A, HSPA1A, HSP90AA1*, and *HSF1* genes in summer have indeed significantly changed (*P* < 0.05). Therefore, they can be considered as key genes that respond to thermal stress and may potentially be used as a reference for marker-assisted selection to develop both high-yielding and thermotolerant Holstein dairy cattle.

EIF2A is an important translation initiation factor and heat stress can activate HRI kinase to phosphorylate EIF2A, phosphorylation EIF2A inhibits EIF2B and prevents the conversion of its bound GDP to GTP, thereby inhibiting the initiation of translation (Figure 6B; Sheikh and Fornace, 1999). Our research shows that in heat-stressed PBMCs, the expression of EIF2A is significantly down-regulated at both the mRNA and protein levels, which may due to the cells reduce protein synthesis by inhibiting the expression of EIF2A to alleviate endoplasmic reticulum stress caused by heat stress. As important genes of the HSP70 and HSP90 families, the important functions of *HSPA1A* and *HSP90AA1* in thermal stress response have been discussed in detail above. Various studies have reported that among all HSP families, the HSP70 and HSP90 families genes are most related to the thermotolerance in sheep (Romero et al., 2013), cattle (Deb et al., 2014), buffalo (Kishore et al., 2014), and goats (Dangi et al., 2014). Heat shock factor 1 (HSF1) is an important transcription factor that plays a central role in heat stress, upregulating proteins such as DNAJB1, HSPA1A, HSP90AA1, and other genes encoding heat shock proteins (Morimoto, 1998). However, in our results, it was found that after heat stress, *HSF1* was not significantly increased as *HSPA1A* and *HSP90AA1* at both the mRNA and protein levels, the possible reasons were that heat stress caused the deficiency of methionine results in the inactivation of HSF1 (Hensen et al., 2012) or its transcriptional activity was regulated by alternative splicing caused by thermal stress (Fujikake et al., 2005; Hensen et al., 2012). HSP90 is a major repressor of HSF1 which can form a stress-sensitive complex with HSF1 (Figure 6B; Zou et al., 1998), and it was also reported that under heat stress condition, HSP70 could suppress the expression of HSF1 in rat myocardial cells (Tang et al., 2016).

It was also observed in our results that some important genes were differentially expressed at the cellular transcriptional and translational levels after thermal stress, due to post-translational regulations such as the regulation of non-coding RNA including miRNA, lncRNA (Smekalova et al., 2016; Panda et al., 2017; Pu et al., 2019). Similar results have also been reported in primary cardiomyocytes subjected to different periods of heat stress (Tang et al., 2016). Moreover, some important genes had different expression trends at the cellular and individual transcription levels, may because individual responses to thermal stress are very complex, although we tried to ensure as much consistency as possible in the background of the experimental individuals, differences inevitably still exist, which is one of the main reasons we chose PBMCs to study thermal stress. Besides we found that several important genes were significantly up-regulated at the cellular level after heat stress, while at the individual level compared with autumn, their expression was significantly lower in summer, similar results have been reported, e.g., cattle *HSPH1* gene expression is higher in autumn than in summer (Sakumoto et al., 2015). This may due to the PBMCs in this study took an acute thermal stress treatment, and the thermal stress produced in the body after individuals experienced a period of chronic heat stress in the summer had not fully recovered to normal levels in the autumn. The specific mechanisms remain to be investigated in further research, and our results would be more convincing if we had also conducted a study of individual protein levels.

## Conclusion

In our present study, the impacts of thermal stress on PBMCs from Holstein dairy cattle were investigated by constructing a cell model, and applying various bioinformatics analysis and molecular biology experimental techniques. Importantly, our results infer that the *HIF1A* gene after cold stress and the *EIF2A*, *HSPA1A*, *HSP90AA1*, and *HSF1* genes after heat stress could be considered as key genes that respond to thermal stress of Holstein dairy cattle. Major biological processes and pathways enriched in response to thermal stress included protein folding and refolding, protein phosphorylation, transcription factor binding, immune effector process, negative regulation of cell proliferation, autophagy, apoptosis, protein processing in endoplasmic reticulum, estrogen signaling pathway, pathways related to cancer, PI3K- Akt signaling pathway, MAPK signaling pathway. The cellular model established in this study with PBMCs provides a suitable platform to improve our understanding of thermal stress in dairy cattle. Furthermore, these key genes and crucial pathways reported in this study could serve as useful references for thermal stress research in dairy cattle and may potentially be used as indicators for marker-assisted selection to develop both high-yielding and thermotolerant Holstein dairy cattle.

## Data Availability Statement

The raw data supporting the conclusions of this article will be made available by the authors, without undue reservation.

## Ethics Statement

The animal study was reviewed and approved by the Committee on Ethics of Animal Experimentation from the Beijing Jiaotong University, Beijing, China. Written informed consent was obtained from the owners for the participation of their animals in this study.

## Author Contributions

QX and YW designed the experiment and supervised the project. LK, LH, and YC conducted the lab work. XT carried out the data analyses. HF wrote the initial manuscript of the manuscript. ZA helped to discuss the results and edit the manuscript. All authors have read and agreed to the published version of the manuscript.

## Conflict of Interest

The authors declare that the research was conducted in the absence of any commercial or financial relationships that could be construed as a potential conflict of interest.

## Footnotes

1 https://www.ncbi.nlm.nih.gov/

2 https://www.genecards.org/

3 http://bioinfo.ut.ee/primer3/

4 http://www.ncbi.nlm.nih.gov/tools/primer-blast/

5 https://david.ncifcrf.gov/

6 http://www.stringdb.org/

7 http://www.cytoscape.org/
