# Supplementary material for: Identification of key Genes and Pathways Associated With Thermal Stress in Peripheral Blood Mononuclear Cells of Holstein Dairy Cattle
Source: Front Genet. 2021 Jun 10;12:662080. doi: 10.3389/fgene.2021.662080 (PMC8222911; doi:10.3389/fgene.2021.662080)
Supplement: Supplementary file 1 [file Data_Sheet_1.docx]

Supplementary Material

**Table S1.** The primers of candidate genes used for qRT-PCR.

| **Number** | **Primer ID** | **Direction** | **Primer sequence (5′‐3′)** | **Amplicon (bp)** |
| --- | --- | --- | --- | --- |
| 1 | *ATF4* | Forward | GCACCAAAACCTCGCAACAT | 107 |
|  |  | Reverse | CTCTCTTCAGCCCCCAAACC |  |
| 2 | *ATF5* | Forward | CTCCACTTTTGCCTTGGTGC | 133 |
|  |  | Reverse | GTCGCCAGGAGTGACATGG |  |
| 3 | *BCL2L11* | Forward | CTGCTGCGTTCGTTCTCTCT | 111 |
|  |  | Reverse | GCCCTCTTGTCTTTTGCTGTTT |  |
| 4 | *CAT* | Forward | CACAGGACCCACTTCTCTGG | 123 |
|  |  | Reverse | CTCACACAGGCGTTTCCTCT |  |
| 5 | *CCDC47* | Forward | GCTAGGCAGTTGAAATAGCAATGAA | 107 |
|  |  | Reverse | TGTAGACACAGAAGAGAACACCAA |  |
| 6 | *CDK9* | Forward | CACCCAGCAGTCTACCAACC | 111 |
|  |  | Reverse | CAAACACGGCCCTAACGAGA |  |
| 7 | *CIRP* | Forward | AGCCCGTCGCCTATGTTTT | 131 |
|  |  | Reverse | ACGTGACTTCCACGCTTTCT |  |
| 8 | *CTSS* | Forward | ATGAACTGGCTGGTGTGGG | 146 |
|  |  | Reverse | CGCCGTGCTACTTCCTCATT |  |
| 9 | *DNAJA1* | Forward | CCTTCTTTTGCTGTGCCATCTT | 131 |
|  |  | Reverse | CCTCTAAGTTTCCCTGCTCCAG |  |
| 10 | *DNAJA3* | Forward | GTTCCAAAGAGACTGACAAGCC | 118 |
|  |  | Reverse | TGGACCGTTTTCCAGTGCTT |  |
| 11 | *DNAJA4* | Forward | ACCGAGGATACCGGAGACAA | 129 |
|  |  | Reverse | GTCTGGGTGGTACTTGAGCG |  |
| 12 | *DNAJB1* | Forward | GGGGCAATGGGAAAGGACTAT | 113 |
|  |  | Reverse | TTCTTGTCCGGGTGGTAACG |  |
| 13 | *DNAJB2* | Forward | GCCGGAGGGAGTCTAGTCA | 108 |
|  |  | Reverse | AGTCAGACCCGAAAGAACCG |  |
| 14 | *DNAJB4* | Forward | TCAAGGCACTCGAAATGGGG | 105 |
|  |  | Reverse | ATCTGAGGGCTTGTTTTCGGT |  |
| 15 | *DNAJB9* | Forward | CCTAAAAGCTCACCCAGTTCCT | 108 |
|  |  | Reverse | AACTGATTGGGGAGTAGCCATTT |  |
| 16 | *DNAJC5* | Forward | CTCAGCAACTTCTGGTCGGT | 127 |
|  |  | Reverse | ATGGTCCATGAACCCGCATT |  |
| 17 | *DNAJC11* | Forward | GTGTGGGTGAAGAGAAGAACCT | 109 |
|  |  | Reverse | CTGTGAGACTGCTTTGGTATCCT |  |
| 18 | *DNAJC17* | Forward | TCCAGTAAGAAGGCAGGCAC | 122 |
|  |  | Reverse | CGTCCCTCCAACCAGGAAAT |  |
| 19 | *DNAJC30* | Forward | CTCCTCTGTTCCTGACTGGTG | 125 |
|  |  | Reverse | GAGTTCCTACTGCCTCACAGC |  |
| 20 | *EIF2A* | Forward | GTCACTAAAAAGGGACTGCTGC | 132 |
|  |  | Reverse | GGGTATCCCAGCCGTATCATC |  |
| 21 | *EPHX2* | Forward | GGAGGACGTGTTTGAGCAGA | 122 |
|  |  | Reverse | CTTGTCCTTGGCTGAGGGAG |  |
| 22 | *ERO1L* | Forward | GCTTCTGGTCAAGGTAAAAGTGAAG | 108 |
|  |  | Reverse | GCTTGCATGGAGGCCAGATA |  |
| 23 | *ERP44* | Forward | GAGAGAATACAGGGGTCAGCG | 111 |
|  |  | Reverse | TCAGGAGTGGTGATTTCTGCC |  |
| 24 | *GAA* | Forward | CAGCGTTCCTGTCTCCAACT | 146 |
|  |  | Reverse | ACTGGGAGGTCTGTGAAGGA |  |
| 25 | *GAPDH* | Forward | GGCGCCAAGAGGGTCAT | 109 |
|  |  | Reverse | AGGCATTGCTGACAATCTTGAG |  |
| 26 | *GBA2* | Forward | AGAGTGAGCCGTGTGAACTG | 139 |
|  |  | Reverse | GGTAGGTAGAAGGGAGGGCA |  |
| 27 | *GSK3B* | Forward | TCACCACTCAAGATGCTAATGCT | 118 |
|  |  | Reverse | ACTTTCACCAAAGGAGGCACT |  |
| 28 | *HIF1A* | Forward | GCCGGGGGAGAACTATGAAC | 143 |
|  |  | Reverse | ACAAATCAGCACCAAGCACG |  |
| 29 | *HMOX1* | Forward | GTAGCCCTCCCACCTCCT | 104 |
|  |  | Reverse | CTGCGGCTGTGTAGGTGT |  |
| 30 | *HSF1* | Forward | ACCTGCAGACCATGCTGAC | 120 |
|  |  | Reverse | ATGCTGGCCAGGCTGC |  |
| 31 | *HSP90AA1* | Forward | GTGGAGACTTTCGCCTTCCA | 131 |
|  |  | Reverse | CTGATCTTGTCCAGGGCGTC |  |
| 32 | *HSP90AB1* | Forward | TCCTCTGCTGATGTCTAGTGGT | 106 |
|  |  | Reverse | ACACAACATTCAAACCCCATGTC |  |
| 33 | *HSPA1A* | Forward | CCTTCACCGATACCGAGCG | 147 |
|  |  | Reverse | AAGGCCACTCCTTCATGTCC |  |
| 34 | *HSPA1L* | Forward | ACCGTATCAAACTGGATCGAAGG | 140 |
|  |  | Reverse | GATCTCCACCTTGCCGTGTT |  |
| 35 | *HSPA2* | Forward | ACGCAAGCACAAGAAGGACA | 128 |
|  |  | Reverse | AGAAATCCACGCCCTCGTAG |  |
| 36 | *HSPA4L* | Forward | AGTAGAGGTTGCTGAAGACAAGG | 112 |
|  |  | Reverse | ATGCTGTGAGTTGTCTTTTGTTGA |  |
| 37 | *HSPA5* | Forward | GTGCCCACCAAGAAGTCTCA | 101 |
|  |  | Reverse | TTGTCTTTCGTCAGGGGTCG |  |
| 38 | *HSPA6* | Forward | TCTACCCGAATCCCCAAGGT | 100 |
|  |  | Reverse | CCCCATAAGCCACAGCTTCA |  |
| 39 | *HSPB2* | Forward | ACGTGCATGGTCACAGTGTAT | 110 |
|  |  | Reverse | CTGAGATTTGGGTTTATTCAGCTCC |  |
| 40 | *HSPB8* | Forward | TAAAATGTTGGGGGTCGGGG | 102 |
|  |  | Reverse | TGCAATGGAGCATGACCTGT |  |
| 41 | *HSPD1* | Forward | CCTGTCTCGTCACCCGC | 117 |
|  |  | Reverse | AAGCCCGAGTGAGATGAGGA |  |
| 42 | *HSPH1* | Forward | CCAGATGCCGAGGCTAATGA | 121 |
|  |  | Reverse | CTTTCCCTAACTGCCAGACCA |  |
| 43 | *HTT* | Forward | CACCGACTACCTCCTCTCCA | 116 |
|  |  | Reverse | GCGGGTAGTTCTCGATCAGG |  |
| 44 | *KDELR1* | Forward | CCGCACGCTCTATCTCTTCA | 109 |
|  |  | Reverse | GAAGTCGCAGTAGAGGACCG |  |
| 45 | *MGAT2* | Forward | TGACGTGGCTGACAAGGTAG | 150 |
|  |  | Reverse | TGAAGGGTCCAGTCCCAGTT |  |
| 46 | *MYC* | Forward | TGTAGTAATTCCAGCGAGAGGC | 125 |
|  |  | Reverse | CTTTGACTCCGGATCTCCCTTC |  |
| 47 | *PIK3C3* | Forward | CGCCTGTGTTGTGCTGTTC | 118 |
|  |  | Reverse | ATAGTGAAACTTCTCCGCTTCCC |  |
| 48 | *PIK3IP1* | Forward | AGCCAGTGATTGGGATCAGC | 121 |
|  |  | Reverse | CCAGCTCCAATGACGACGAT |  |
| 49 | *PIK3R1* | Forward | ACAACTCACGGCTCAAGAAGA | 122 |
|  |  | Reverse | TACCAGCCTTAACACAGCCC |  |
| 50 | *RAC1* | Forward | GTCCTACCCGCAGACAGATG | 144 |
|  |  | Reverse | TCAAGTTTCGTCCCCACCAG |  |
| 51 | *SLC38A2* | Forward | TTGTCCGTTTGGCTGTGTTG | 119 |
|  |  | Reverse | ATGACGCCACCAACTGAACT |  |
| 52 | *SOD1* | Forward | CACCATCCACTTCGAGGCAA | 102 |
|  |  | Reverse | TCTCCAAACTGATGGACGTGG |  |
| 53 | *STK4* | Forward | CCTGAAGAGTTGGACGGTGG | 102 |
|  |  | Reverse | TTCGACTGGTACTTCTGCCG |  |
| 54 | *STT3B* | Forward | GCACTATTTGGGGGACGACA | 114 |
|  |  | Reverse | TTCCTCACTTTGCCTGCCTT |  |
| 55 | *TGFB1* | Forward | GAACTGCTGTGTTCGTCAGC | 121 |
|  |  | Reverse | GCTCCAGATGTAAGGGCAGG |  |
| 56 | *VCP* | Forward | AGGGGAGCCAATCAAACGAG | 119 |
|  |  | Reverse | TCAAGGGCAACTCCACCATC |  |

**Table S2.** The expression changes of candidate genes at the cellular transcription level by using qRT-PCR analysis.

| **Genes** | **Cold stress group (25 ℃)** | | **Heat stress group (42 ℃)** | |
| --- | --- | --- | --- | --- |
|  | **Fold Change** | ***P*-Value** | **Fold Change** | ***P*-Value** |
| *ATF4* | 0.68 | 3.75E-05 | 0.69 | 9.94E-04 |
| *ATF5* | 0.83 | 9.59E-02 | 1.97 | 1.38E-04 |
| *BCL2L11* | 1.00 | 7.72E-01 | 8.33 | 3.48E-05 |
| *CAT* | 0.59 | 2.79E-06 | 0.40 | 1.37E-08 |
| *CCDC47* | 0.59 | 3.28E-04 | 0.43 | 3.58E-06 |
| *CDK9* | 0.72 | 8.81E-03 | 0.53 | 1.88E-03 |
| *CIRP* | 1.25 | 1.09E-02 | 0.39 | 3.11E-07 |
| *CTSS* | 0.96 | 4.07E-01 | 0.92 | 3.25E-01 |
| *DNAJA3* | 0.72 | 1.12E-03 | 0.56 | 2.70E-04 |
| *DNAJB1* | 1.00 | 8.60E-06 | 90.80 | 1.52E-05 |
| *DNAJB2* | 0.57 | 6.17E-03 | 0.72 | 3.27E-02 |
| *DNAJB4* | 0.56 | 1.64E-03 | 23.72 | 1.31E-04 |
| *DNAJC11* | 0.80 | 3.44E-05 | 0.38 | 2.13E-05 |
| *DNAJC17* | 0.64 | 1.15E-06 | 0.41 | 1.64E-05 |
| *DNAJC30* | 0.71 | 5.27E-05 | 0.30 | 9.85E-07 |
| *DNAJC5* | 0.85 | 1.43E-02 | 0.80 | 6.44E-03 |
| *EIF2A* | 0.82 | 1.24E-03 | 0.56 | 5.06E-09 |
| *EPHX2* | 1.08 | 4.09E-01 | 1.09 | 4.59E-01 |
| *ERO1L* | 0.88 | 2.50E-02 | 0.42 | 3.84E-06 |
| *ERP44* | 0.68 | 2.46E-03 | 0.32 | 5.23E-06 |
| *GAA* | 1.20 | 2.43E-02 | 0.88 | 8.66E-02 |
| *GBA2* | 0.82 | 2.19E-03 | 0.73 | 2.36E-04 |
| *GSK3B* | 1.02 | 4.62E-01 | 0.94 | 9.76E-02 |
| *HIF1A* | 1.41 | 2.11E-03 | 0.92 | 4.91E-02 |
| *HSF1* | 0.97 | 4.65E-01 | 0.61 | 8.80E-06 |
| *HSP90AA1* | 0.77 | 4.73E-03 | 12.23 | 7.69E-04 |
| *HSP90AB1* | 0.75 | 3.29E-03 | 3.01 | 7.18E-04 |
| *HSPA1A* | 1.11 | 5.57E-01 | 320.00 | 3.41E-05 |
| *HSPA2* | 0.87 | 4.92E-01 | 4.06 | 1.86E-06 |
| *HSPA4L* | 1.09 | 4.73E-01 | 11.64 | 2.71E-05 |
| *HSPA5* | 1.03 | 5.65E-01 | 1.70 | 4.14E-03 |
| *HSPB2* | 0.64 | 7.60E-04 | 2.17 | 1.47E-03 |
| *HSPB8* | 0.50 | 7.60E-04 | 43.00 | 5.48E-05 |
| *HSPD1* | 0.88 | 8.80E-01 | 2.37 | 5.46E-03 |
| *HSPH1* | 0.73 | 3.80E-05 | 1.67 | 4.17E-03 |
| *HTT* | 0.56 | 2.17E-03 | 0.45 | 3.64E-04 |
| *KDELR1* | 0.93 | 6.78E-02 | 0.83 | 6.46E-04 |
| *MGAT2* | 0.71 | 4.42E-04 | 0.65 | 9.86E-07 |
| *MYC* | 0.69 | 2.04E-02 | 0.42 | 1.77E-03 |
| *PIK3C3* | 1.15 | 2.03E-02 | 0.64 | 4.54E-03 |
| *PIK3IP1* | 0.43 | 1.08E-03 | 0.12 | 1.97E-04 |
| *PIK3R1* | 0.49 | 2.12E-05 | 0.54 | 7.13E-05 |
| *RAC1* | 0.87 | 9.35E-02 | 0.60 | 1.32E-03 |
| *SLC38A2* | 0.66 | 5.18E-06 | 1.66 | 2.36E-06 |
| *SOD1* | 0.90 | 5.41E-01 | 0.98 | 8.67E-01 |
| *STK4* | 0.61 | 2.39E-02 | 0.73 | 7.97E-02 |
| *STT3B* | 0.52 | 1.58E-02 | 0.84 | 3.46E-01 |
| *TGFB1* | 0.93 | 3.10E-01 | 0.79 | 9.76E-03 |
| *VCP* | 0.95 | 2.16E-01 | 0.82 | 2.97E-03 |

**Table S3**. KEGG pathway enrichment analysis (Top 6) for differently expressed genes (DEGs) after thermal stress.

| **Treatment Group** | **Pathways** | **Count** | ***P*-Value** | **Genes** |
| --- | --- | --- | --- | --- |
| Cold stress (25 ℃) | Protein processing in endoplasmic reticulum | 6 | 3.58E-05 | *STT3B*, *HSP90AB1*, *HSPH1*, *ATF4*, *HSP90AA1*, *DNAJB2*, |
|  | Prostate cancer | 4 | 1.03E-03 | *HSP90AB1*, *ATF4*, *HSP90AA1*, *PIK3R1* |
|  | Estrogen signaling pathway | 4 | 1.50E-03 | *HSP90AB1*, *ATF4*, *HSP90AA1*, *PIK3R1* |
|  | Pathways in cancer | 6 | 1.92E-03 | *HSP90AB1*, *HSP90AA1*, ***HIF1A***, *STK4*, *MYC*, *PIK3R1* |
|  | PI3K-Akt signaling pathway | 5 | 8.04E-03 | *HSP90AB1*, *ATF4*, *HSP90AA1*, *MYC*, *PIK3R1* |
|  | Central carbon metabolism in cancer | 3 | 9.33E-03 | ***HIF1A****, MYC, PIK3R1* |
| Heat stress (42 ℃) | Protein processing in endoplasmic reticulum | 12 | 2.39E-13 | ***HSP90AB1***, ***HSPH1***, *ATF4*, ***HSP90AA1***, *VCP*, ***HSPA2***, ***HSPA4L***, *DNAJB2*, ***HSPA1A***, *DNAJC5*, ***DNAJB1***, ***HSPA5*** |
|  | Estrogen signaling pathway | 6 | 1.23E-06 | ***HSP90AB1***, *ATF4*, ***HSP90AA1***, ***HSPA2***, ***HSPA1A***, *PIK3R1* |
|  | Legionellosis | 5 | 1.77E-06 | *VCP*, ***HSPA2***, *HSF1*, ***HSPA1A***, ***HSPD1*** |
|  | Antigen processing and presentation | 4 | 1.54E-04 | ***HSP90AB1***, ***HSP90AA1***, ***HSPA2***, ***HSPA1A*** |
|  | MAPK signaling pathway | 6 | 2.82E-04 | *ATF4*, ***HSPA2***, *RAC1*, ***HSPA1A***, *MYC*, *TGFB1* |
|  | PI3K-Akt signaling pathway | 7 | 1.44E-03 | ***HSP90AB1****, ATF4,* ***HSP90AA1****, RAC1, MYC,* ***BCL2L11****, PIK3R1* |

**Note:** Genes up-regulated by acute cold or heat stress were shown in bold and down-regulated genes were shown in normal.
